# Surgical anatomy of the ovine sural nerve for facial nerve regeneration and reconstruction research

**DOI:** 10.1038/s41598-019-46661-3

**Published:** 2019-07-22

**Authors:** Yosuke Niimi, Satoshi Fukuda, Ryan S. Gilbert, Tuvshintugs Baljinnyam, Yu Niimi, Hajime Matsumine, Keibun Liu, Sam Jacob, Hal K. Hawkins, Robert A. Cox, David N. Herndon, Donald S. Prough, Perenlei Enkhbaatar

**Affiliations:** 1 Department of Anesthesiology, University of Texas Medical Branch, Galveston, Texas 77555 USA; 2 Department of Plastic and Reconstructive Surgery, Tokyo Women’s Medical University, Shinjuku-ku, Tokyo 162-8666 Japan; 3 Department of Pathology, Shriners Hospitals for Children, Galveston, Texas 77550 USA; 4 Center for Multiphasic Health Testing and Services, Mitsui Memorial Hospital, Chiyoda-ku, Tokyo 101-8043 Japan; 5 Department of Pathology, University of Texas Medical Branch, Galveston, Texas 77555 USA; 6 Department of Surgery, Shriners Hospitals for Children, Galveston, Texas 77550 USA; 7 Shriners Hospital for Children, Galveston, Texas 77550 USA

**Keywords:** Neurology, Anatomy, Neurological disorders

## Abstract

The lack of a clinically relevant animal models for research in facial nerve reconstruction is challenging. In this study, we investigated the surgical anatomy of the ovine sural nerve as a potential candidate for facial nerve reconstruction, and performed its histological quantitative analysis in comparison to the buccal branch (BB) of the facial nerve using cadaver and anesthetized sheep. The ovine sural nerve descended to the lower leg along the short saphenous vein. The length of the sural nerve was 14.3 ± 0.5 cm. The distance from the posterior edge of the lateral malleolus to the sural nerve was 7.8 ± 1.8 mm. The mean number of myelinated fibers in the sural nerve was significantly lower than that of the BB (2,311 ± 381vs. 5,022 ± 433, respectively. p = 0.003). The number of fascicles in the sural nerve was also significantly lower than in the BB (10.5 ± 1.7 vs. 21.3 ± 2.7, respectively. p = 0.007). The sural nerve was grafted to the BB with end-to-end neurorrhaphy under surgical microscopy in cadaver sheep. The surgical anatomy and the number of fascicles of the ovine sural nerve were similar of those reported in humans. The results suggest that the sural nerve can be successfully used for facial nerve reconstruction research in a clinically relevant ovine model.

## Introduction

Many patients suffer from defects of the buccal branch of the facial nerve caused by malignant craniofacial tumors or various traumas, including burns. This becomes a serious long-term issue because the buccal branch is responsible for many vital activities such as facial expression, eating, and drinking. Autologous sural nerve grafting with an end-to-end neurorrhaphy technique to bridge defects of the buccal branch is the gold standard of surgical treatment for facial nerve reconstruction^1^. The sural nerve is frequently used as a donor nerve for human facial nerve reconstruction because it is a sensory nerve, can be easily harvested, and is associated with relatively low donor site morbidity^2,3^.

There are many basic science preclinical models in rats that are currently used for the development of novel techniques for facial nerve reconstruction and regeneration, including the use of various scaffolds^4,5^, stem cells^6,7^, and neurorrhaphy techniques^8^. The pathophysiology of facial palsy has been studied in rat models as well^9,10^. However, most of the rodent studies have used the sciatic or great auricular nerves because these nerves are harvested easily, and the rodent sural nerve is too short. For this reason, a clinically-relevant large animal nerve graft model for facial nerve regeneration research is needed. Such a model may offer a better translational approach in this field.

Sheep are frequently used to mimic clinical scenarios of human disease and treatment because the anatomy of their organs (e.g. lung, spine, skin, and subcutaneous tissue), as well as their responses to inflammation, are similar to those in humans^11–14^. We have previously reported the surgical anatomy of the ovine facial and hypoglossal nerves, and described anatomical and histological similarities of these nerves to their human counterparts^15^. By contrast, the anatomy and functional capacity of these nerves in rodents and swine are quite different from those in humans^16,17^. May *et al*. partially described the anatomy of the ovine sural nerve, but in that work sural nerve harvesting surgical technique was not sufficiently described, and histological assessments were not performed^18^. The goal of this study was to establish an ovine model of facial nerve grafting for future research on facial nerve regeneration and reconstruction. We have investigated the surgical anatomy of the ovine sural nerve and its related nerves (i.e., medial sural cutaneous nerve (MSCN), tibial, common fibular, and sciatic nerves), and compared the histological structures of these nerves to those of the buccal branch.

## Results

### Surgical procedure to identify the ovine sural nerve

**Figure 1:**
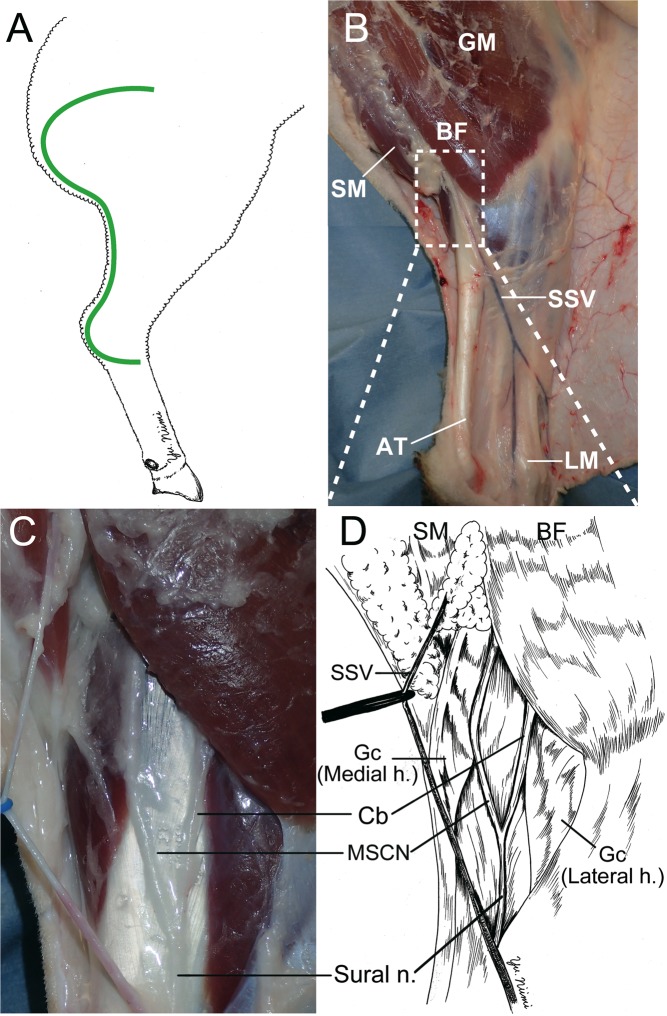
Surgical procedure to identify of the ovine sural nerve. (**A**) The green line shows the incision line. From the lateral gluteal-thigh border to 2 cm posterior the lateral malleolus C-shape incision was made on the ovine cadaver right leg. (**B**) The gluteus maximus (GM), biceps femoris (BF), semimembranosus (SM), Achilles tendon (AT), lateral malleolus (LM) were dissected after undermining the skin flap. The short saphenous vein (SSV) was identified. (**C**,**D**) The sural nerve, medial sural cutaneous nerve (MSCN), and communicating branch (Cb) were identified to be located under the gastrocnemius fascia, between the lateral and medial head of the gastrocnemius, and distal of BF. Sural nerve formed by MSCN and Cb. Gc (Medial h.): Gastrocnemius medial head. Gc (Lateral h.): Gastrocnemius lateral head.

C-shaped, full-thickness skin incision from the lateral gluteal-thigh border to 2 cm posterior to the lateral malleolus was made on the ovine cadaver right leg (Fig. 1A), and full-thickness skin was undermined widely using a scalpel. The gluteal muscle, biceps femoris muscle, semimembranosus muscle, Achilles tendon, and short saphenous vein were identified (Fig. 1B). The sural nerve, the medial sural cutaneous nerve (MSCN), and a communicating branch were found to be located under the gastrocnemius fascia, between the lateral and medial heads of the gastrocnemius, distal to the biceps femoris (Fig. 1C,D). The sural nerve was formed by the MSCN and communicating branch, and descended to the distal portion.

### Surgical anatomy of the ovine sural nerve and its related nerves

**Figure 2:**
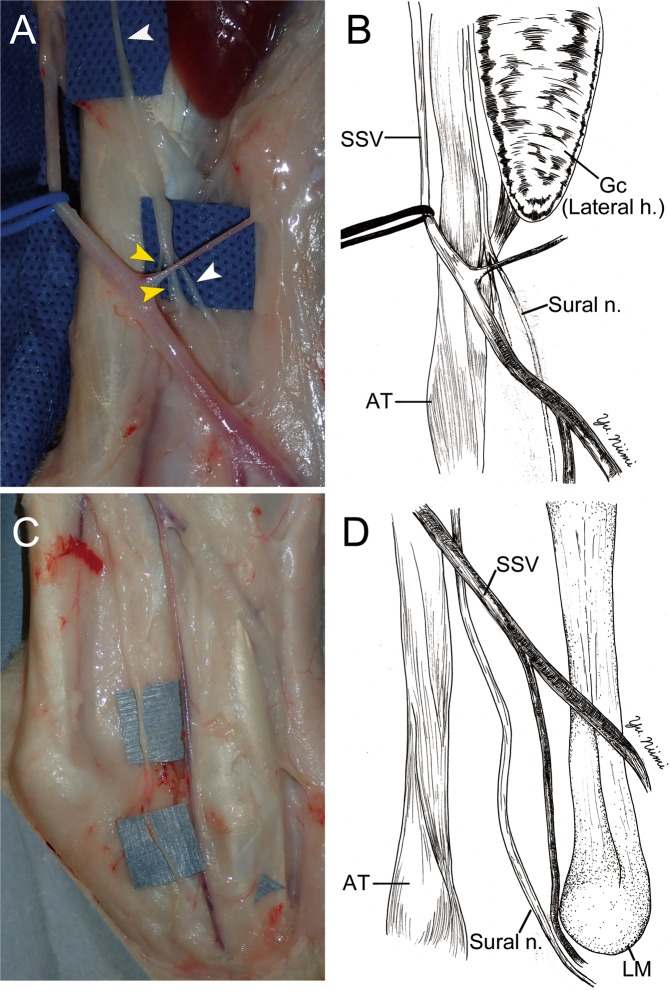
The surgical anatomy of sural nerve (Sural n.); middle (**A**,**B**) and distal (**C**,**D**) portion of the leg. (**A**,**B**) The sural nerve (white arrow head) ran parallel to the short saphenous vein (SSV, blue rubber tape), and ran medial side of the gastrocnemius (Gc) lateral head (Lateral h.). Thereafter, two nerves (yellow arrow heads) were merging to the sural nerve at the midpoint of the lower leg. (**C**,**D**) In the distal portion of lower leg, the sural nerve was passed posterior to the lateral malleolus (LM). Thereafter, the sural nerve penetrated the deep fascia and went into the foot area. AT: Achilles tendon. Gc (Lateral h.): Gastrocnemius lateral head.

The sural nerve descended in the gastrocnemius fascia pararell to the short saphenous vein. Thereafter, the sural nerve descended into the subcutaneous tissue approximately at the midpoint of the lower leg. (Fig. 2A,B). The sural nerve descended along with the short saphenous vein to the distal portion of the lower leg. The sural nerve ran posterior to the lateral malleolus in the subcutaneous tissue. After passing over the lateral malleolus, the sural nerve went into deep subcutaneous tissue (Fig. 2C,D).

**Figure 3:**
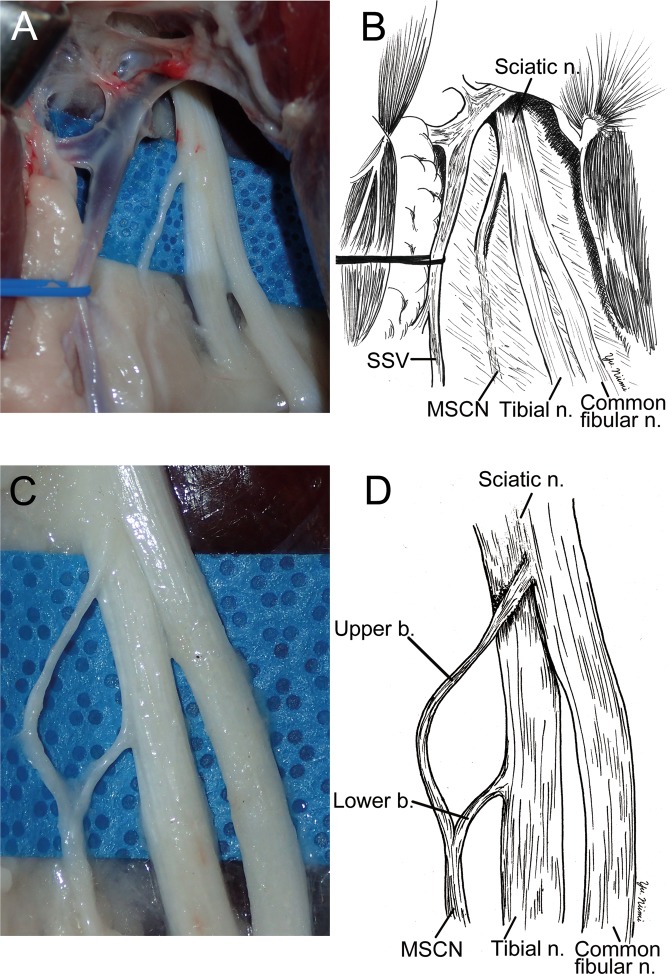
The surgical anatomy of medial sural cutaneous nerve (MSCN). (**A**,**B**) To dissect to the proximal region from the point of Fig. 1, MSCN was divided from sciatic nerve (Sciatic n.) near the tibial nerve side between biceps femoris and semimembranosus. Common fibular nerve (Common fibular n.) was also devided from scaiatic nerve at the same place. (**C**,**D**) After removing connective tissue, MSCN was exposed forming from two branches, which were originated from sciatic and tibial nerves.

The medial sural cutaneous nerve (MSCN) was dissected to the popliteal fossa. The MSCN, tibial nerve, common fibular nerve, and sciatic nerve were identified between the biceps femoris and semimembranosus muscles (Fig. 3A,B). To identifiy the detailed anatomy of the MSCN, the surounding tissue menbrane was removed. The MSCN formed two branches. One branch, originating from the sciatic nerve, was located between the tibial and common fibular nerves. The other branch originated from the tibial nerve (Fig. 3C,D). The MSCN descended pararell to the short saphenous vein in the gastrocnemius fascia. Thereafter, the MSCN joined with a communicating branch and formed the sural nerve in the fascia.

### Dimensions of the sural nerve and buccal branch

**Figure 4:**
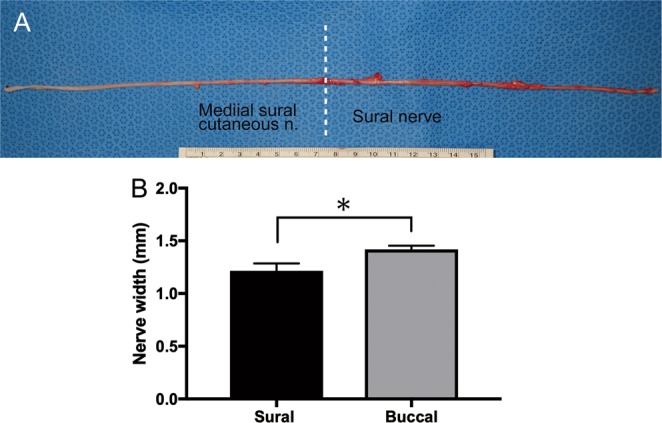
(**A**) The picture of sural nerve and medial sural cutaneous nerve. The average lengths of the medial sural cutaneous and sural nerve were 14.3 ± 0.5 (n = 7) and 16.4 ± 0.8 cm (n = 7), respectively. The average of total length was 30.2 ± 0.7 cm (n = 16). Unit of the ruler: cm (**B**) The width of sural nerve (n = 10) and buccal branch (n = 8) were measured using the photographs by ImageJ software version 1.50 (National Institutes of Health, Bethesda, MD) The width of the sural nerve was significantly lower than that of the buccal branch (1.2 ± 0.07 vs. 1.4 ± 0.04 mm respectively. p = 0.0117).

**Figure 5:**
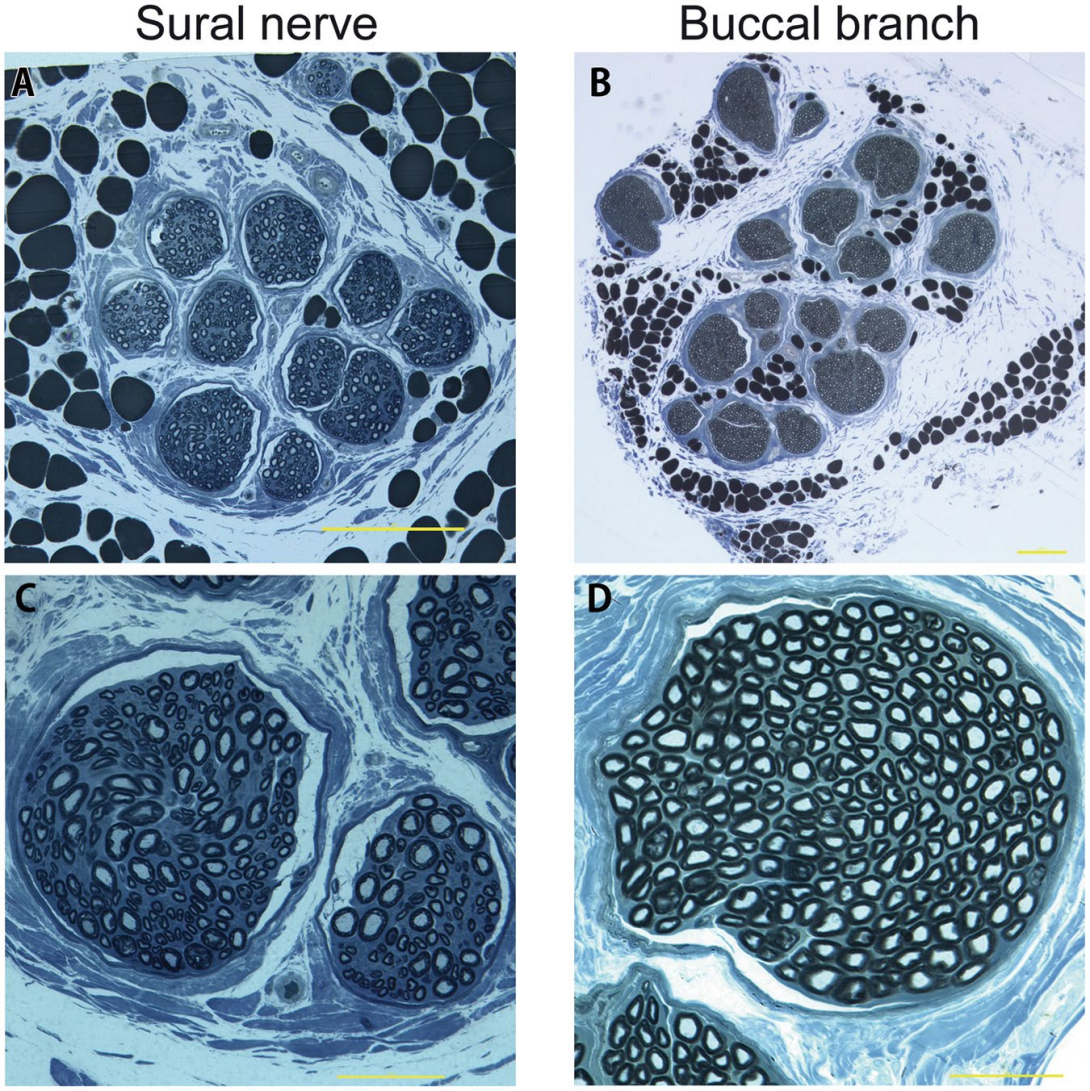
The toluidine blue stain of sural nerve (Left column) and buccal branch (right column). Both sural nerve (**A**) and buccal branch (**B**) had epineurium. (**C**) Myelin sheaths of axons of the sural nerve was stained blue color and observed clearly. (**D**) Dense myelin sheath of buccal branch was observed. (**A**,**B**) Scale bar: 200 μm. (**C**,**D**) Scale bar: 50 μm.

The sural nerve from the proximal to the distal end was harvested (Fig. 4A). The length of the sural nerve and MSCN were 14.3 ± 0.5 and 16.4 ± 0.8 cm, respectively (Table 1). The distance from the posterior edge of the lateral malleolus to the sural nerve was 7.8 ± 1.8 mm (Table 1). The width of the sural nerve was significantly lower than that of the buccal branch of the facial nerve (1.2 ± 0.07 vs. 1.4 ± 0.04 mm respectively. p = 0.0117) (Fig. 5B).

**Table 1:** The dimensions of the sural nerve and medial sural cutaneous nerve (MSCN).

Measurement items	This study	Human
Length of the sural nerve (cm)	14.3 ± 0.5	11–20^21,22^
Length of the MSCN (cm)	16.4 ± 0.8	21–33^21^
Distance from the posterior of lateral malleolus to the sural nerve (mm)	7.8 ± 1.8	7 ± 5^19^
Diameter of the sural nerve (mm)	1.2 ± 0.07	2.0–3.0^21^ 3.5–3.8^20^
Diameter of the buccal branch (mm)	1.4 ± 0.04	Not stated

Data in this study are shown as mean ± SEM.

### No functional motion of the leg was observed after stimulating the sural nerve and the MSCN

By electric stimulation, we determined that the common fibular and tibial nerves innervated leg muscles. On the other hand, no innervation of leg muscles was observed after stimulation of the sural nerve or the MSCN. These results showed that the sural nerve and the MSCN are sensory nerves.

### Histological assessments of sural nerve and buccal branch

**Figure 6:**
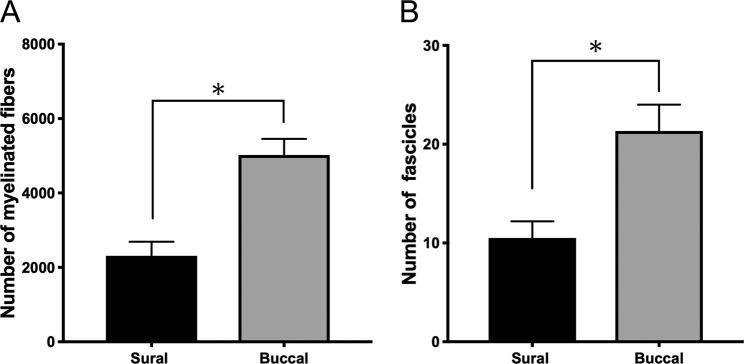
Histological assessment. (**A**) The mean number of myelinated fibers in the Sural nerve were significantly lower than that in the buccal branch (2,311 ± 381 vs. 5,022 ± 433, respectively, p = 0.003). (**B**) The number of fascicles in the sural nerve were significantly lower than that in the buccal branch (10.5 ± 1.7 vs. 21.3 ± 2.7, respectively, p = 0.007).

In semithin sections of plastic-embedded specimens stained to provide dark blue staining of myelin, sections of both the sural nerve and the buccal branch of the facial nerve revealed multiple fascicles surrounded by epineurium. Small peripheral blood vessels were observed in both nerves (Fig. 5). The mean number of myelinated fibers in the sural nerve was significantly lower than in the buccal branch (2,311 ± 381 vs. 5,022 ± 433, respectively, p = 0.003) (Fig. 6A). The number of fascicles in the sural nerve was significantly lower than in the buccal branch (10.5 ± 1.7 vs. 21.3 ± 2.7, respectively, p = 0.007) (Fig. 6B).

### Sural nerve graft to the buccal branch with end-to-end neurorrhaphy

**Figure 7:**
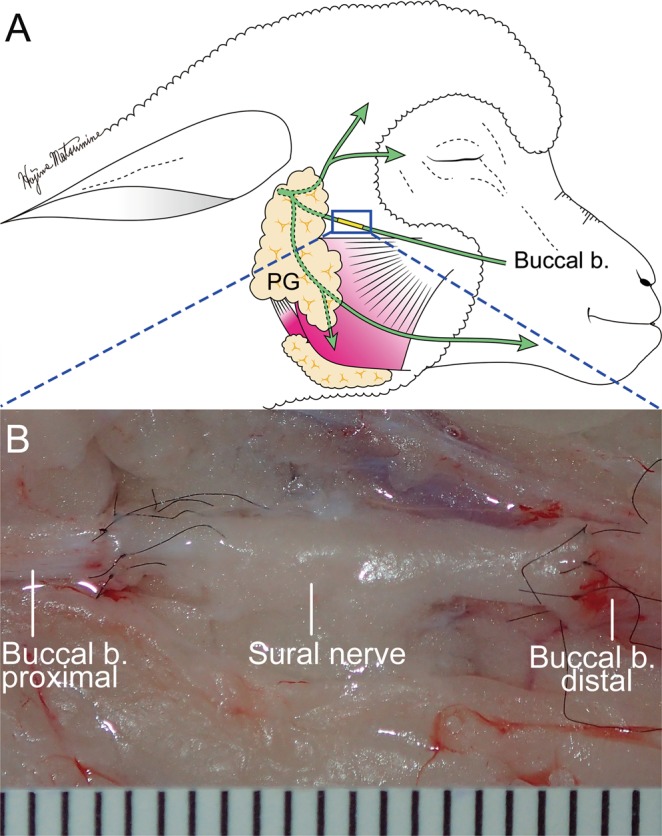
Demonstration of sural nerve graft to the buccal branch with end-to-end neurorrhaphy. (**A**) Schematic diagram of sural nerve graft. end-to-end neurorrhaphy was performed 10 mm distal to the edge of parotid gland (PG). (**B**) Microscopical image of end-to-end neurorrhaphy. Fifteen millimeter-sural nerve was grafted 6 perineural sutures using 10-0 nylon to bridge the defect of buccal branch with end-to-end neurorrhaphy under the surgical microscope. One unit indicates 1 mm.

A 15-mm segment of the sural nerve was grafted with six-epineural suture techniques using 10-0 nylon to bridge the defect in the buccal branch of the ovine facial nerve with end-to-end neurorrhaphy under a surgical microscope. Although the diameter of the donor (sural) nerve was smaller compared to the recipient (buccal) nerve, epineural suturing was performed easily without any tension (Fig. 7).

## Discussion

In this study, we aimed to describe the surgical anatomy of the ovine sural nerve. Additionally, the surgical anatomy of the medial sural cutaneous nerve (MSCN) was determined. We found some similarities of sural nerve between sheep and humans, as follows: (1) The ovine sural nerve descended along with the short saphenous vein between the medial and lateral heads of the gastrocnemius muscle, similarly to humans; (2) The distal part of the ovine sural nerve was found to be located at an average of 7.8 ± 1.8 mm from the posterior edge of the lateral malleolus, similar to the 7 ± 5 mm distance in humans^19^. This finding was usefull as a landmark to identify the ovine sural nerve when it was harvested. As the anatomical variations in human sural nerve are well documented^20,21^, further investigations of variations of the ovine sural nerve anatomy and pathways are needed; (3) The length of the ovine sural nerve (14.3 cm) was in the same range as in humans (11–20 cm)^21^. In humans, a maximum 40 cm of sural nerve can be harvested including MSCN^22^. In our ovine study, an average of 30 cm of nerve could be harvested including MSCN. This enables studies requiring long donor nerves to perform various grafts such as an interpositional jump graft or cross face nerve graft^3,23,24^. The length of the sural nerve in the miniature pig (from the sciatic nerve to its distal end) is 12.3 ± 1.2 cm^25^; and (4) Electric nerve stimulation proved that the sural nerve is a sensory nerve similar to that in humans. In addition, the human sural nerve innervates the dorsal lateral foot. Further studies are warranted to explore the sensory area of ovine sural nerve innervation, and examine the possibility of long-term complications such as donor site decubitus.

In our study, we found some differences of sural nerve anatomy and morphology between ovine and human as follows: (1) The ovine sural nerve penetrated the fascia and soon naturally went into the subcutaneous tissue at approximately half of the length of the lower leg, because ovine MSCN joined a communicating branch before going into subcutaneous tissue, whereas 84% of human MSCN join the communicating branch after the MSCN penetrates the crural fascia in the middle third of the leg^26^; and (2) the number of myelinated fibers in ovine sural nerve was 2,311 ± 381. Although we found that the number of fascicles in the sural nerve in sheep was in same range as in humans (9–14)^21^, the number of myelinated fibers in the sural nerve in sheep seemed to be lower than in humans. In humans, the number of myelinated fibers of the sural nerve described by Chentanez^27^, Jacob^28^, and Behse^29^ were 5,672.8 ± 1,753.7, 5,918.3 ± 1,201.9 and 7,252.5 ± 1,242.8, respectively.

Interstingly, we also found that the number of myelinated fibers in the ovine buccal branch (4,768 ± 430) was much higher than in humans (1,670, average age 72 years)^30^. In another study, the number of myelinated nerve fibers in the human buccal branch was reported as 1,736 without indicating the age^31^. Although the basis for this discrepancy is not known, we speculate the following: (1) It has previously been shown that when age increases, the number of buccal nerve as well as facial nerve trunk fibers decreases^30,32^. In the present study, we used approximately 3-year-old sheep, equivalent to 30-year-old humans; and (2) we counted only myelinated fibers in our ovine study. Jacob *et al*. reported that the total number of myelinated fibers and unmyelinated axons in human buccal branch is 5,589^31^.

In this study, we have also developed a technique for grafting of a segment of sural nerve to the buccal branch of the facial nerve using end-to-end neurorrhaphy in cadaver sheep. Although the ovine sural nerve was narrower than the buccal branch, we were able to successfully perform end-to-end neurorrhaphy without any technical problemsThin cutaneous nerve grafts are easily revascularized leading to successful engraftment^33^. In regard this, the ovine sural nerve can be an excellent candidate for buccal branch reconstruction. In addition, the number of ovine sural myelinated fibers was less than that of ovine buccal branch. Despite numerous reports, the significance of matching the number of donor and recipient nerves remains unclear^34,35^. Terzis *et al*. reported that number of axon of proximal recipient nerves was more correlated with functional outcome than that of a donor nerve in the clinical cross face nerve graft study^36^. Future research is warranted to investigate the degree of grafted sural nerve regeneration.

Artificial nerve conduits are used for treating the sensory nerve, such as digital nerve injuries^37^. In addition, various artificial nerve conduits (polyglycolic acid^5^, poly lactic-co-glycolic acid^38^, collagen^39^, and acellular nerve allografts^40,41^) and cell therapy (adipose derived stem cells^42^, stromal vascular fraction^6^, dental pulp cells^43^, and Schwann cells^44^) have been reported to be beneficial in small animal studies. However, only a few clinical studies reported use of artificial nerve conduits for motor nerve reconstruction including facial nerves^45^. The lack of clinical studies using artificial nerves may be related to the fact that the nerves reconstructed with artificial nerves are less functional compared to that reconstructed with “gold standard” autologous nerve graft^46^. The other reason may be related to the lack of clinically relevant large animal models allowed to investigate the effects of artificial nerve conduits in comparison to autologous nerve grafts. We believe that an ovine model of sural nerve regeneration may fill the existing gap.

The limitations of this study include a relatively small number of animals studied. Another limitation is that this study describes sural anatomy and histology only in the right side of the ovine leg. Additionally, this study did not directly evalaute human buccal and sural nerve anatomy, instead it compared ovine sural nerve anatomy to those in humans that have been previousy published elswhere^27–29^. Also, this study did not focus on functional aspects of the sural nerve and its branches.

In summary, the results of the present study show that the surgical anatomy and morphology of the ovine sural nerve are quite similar to those of humans, suggesting that an ovine sural nerve grafting model can be successfully used for research on facial nerve regeneration and reconstruction. Future research is warranted to study facial nerve regeneration using this clinically-relevant ovine model with end-to-end neurorrhaphy.

## Materials and Methods

For this study, twenty-three cadaver sheep (female Merino sheep, 36.7 ± 0.8 kg, approximately 3 years old; Talley Ranch, Bastrop, TX) and three anesthetized sheep (39.3 kg ± 1.2 kg, approximately 3 years old) were used. All animal studies were conducted in adherence with the guidelines detailed in the NIH Guide for the Care and Use of Laboratory Animals. The study was reviewed and approved by the Institutional Animal Care and Use Committee (IACUC) of the University of Texas Medical Branch, Galveston, TX, USA.

### Experimental design

The surgical anatomy of the sural nerve (n = 23) and the histology of the sural nerve (n = 8) and the buccal branch of the facial nerve (n = 6) were studied in cadaver sheep. These sheep were euthanized after completing other studies (i.e., pulmonary, wound healing) by intravenous injection of saturated potassium chloride under deep anesthesia and analgesia (ketamine 5 mg/kg, xylazine 100 mg and buprenorphine 0.3 mg). In addition, three cadaver sheep were used for neurorrhaphy.

Three anesthetized sheep were used for nerve stimulation. These sheep were pre-medicated with 2.5 mg/kg ketamine, and 2% isoflurane via mask. The sheep were then intubated and anesthesia continued with 2–5% isoflurane and mechanically ventilated.

### Surgical procedure and measured variables of the sural nerve

To investigate the ovine sural nerve anatomy, the cadaver sheep were positioned at right lateral position. The dimensions of sural nerve were measured as follows: the length of the sural nerve from the distal end of the medial sural cutaneous nerve (MSCN) to the end point of the lateral malleolus; the length of the MSCN; the distance between the posterior margin of the lateral malleolus and the sural nerve; and the diameter of the sural nerve at a point 2 cm proximal to the distal end using the photographs by ImageJ software version 1.50 (National Institutes of Health, Bethesda, MD). Then, a 15 mm segment of the sural nerve beginning at a point 5 cm proximal from the lateral malleolus was sharply harvested using a scalpel for histological analysis.

### Surgical procedure to harvest the buccal branch of the facial nerve

The surgical procedure for approaching the buccal branch was described previously^15^. Briefly, a pre-auricular incision extending from 1.5 cm posterior to the mandibular ramus to a submandibular point 2 cm below the mandibular body was made in the right face of the sheep cadaver. The platysma muscle was dissected to expose the buccal branch of the facial nerve and the parotid gland. Thereafter, the diameter of the buccal branch at a point 1 cm distal to the anterior parotid border was measured by ImageJ using the photograph. Then, the 15 mm-buccal branch at the same point was sharply harvested by scalpel for histological analysis.

### Stimulation of the sural nerve and the sciatic nerve and its branches

The same incision as in cadaver study was made on the right leg of anesthetized sheep without use of paralytic agents. After their exposure, the sural nerve and the sciatic nerve and its branches (i.e., MSCN, tibial, and common fibular nerves) were stimulated (approximately 10 mA, 200-μsec monopolar pulses/second for one second) from an electrocautery pencil tip (Rocker Switch Pencil, COVIDIEN, MA, USA) attached to the clamp end of the electrodes supplied with the nerve stimulator (INNERVATOR NS252, Fisher and Paykel Healthcare, New Zealand). The biceps femoris, gastrocnemius, and tibialis anterior muscle contractions were observed and photographed.

### Semithin sections of toluidine blue stain of myelinated fibers

Immediately upon surgical extraction, the sural nerve and buccal nerve branch specimens were placed in a buffered fixative containing formaldehyde and glutaraldehyde for 24 hours (Trump’s Fixative, Electron Microscopy Sciences, PA, USA). Nerve specimens were then post-fixed with 2% osmium tetroxide diluted in 0.1 mol/L cacodylate buffer overnight before being embedded in epoxy resin (EMBed-812 Embedding Kit, Electron Microscopy Sciences, PA, USA). The resin embedded specimens were then cut at 2 μm thickness using an ultramicrotome and stained with 1% toluidine blue. Images of the semithin sections were then captured under a light microscope. Numbers of myelinated fibers and fascicles were manually counted using ImageJ.

### Sural nerve graft to repair a defect of the buccal branch of the facial nerve with end-to-end neurorrhaphy

To perform end-to-end neurorrhaphy, the right lateral incision described above was made in the face of a cadaver sheep. A 15-mm long section of buccal branch was exposed and dissected from the surrounding connective tissues on the fascia of the masseter muscle using micro-scissors at a point 1 cm distal to the anterior parotid border. Then, a 15 mm long nerve defect of the buccal branch was made. A 15 mm long segment of the sural nerve located 5 cm above the lateral malleolus was harvested in the right leg. The sural nerve segment was sutured using 10-0 nylon (Ethilon, Ethicon, NJ) to bridge the defect of the buccal branch with end-to-end neurorrhaphy using a surgical microscope (OPMI-1, ZEISS, Germany).

### Statistical analysis

Numerical data are expressed as means ± SEM. Data values in two groups were compared by unpaired t-test. The probability of less than 0.05 (*P* < 0.05) was considered significant. Statistical analysis was performed with GraphPad Prism version 7.00 (GraphPad Software, La Jolla, CA.).
